# Autophagy and Cancer Dormancy

**DOI:** 10.3389/fonc.2021.627023

**Published:** 2021-03-19

**Authors:** Yunus Akkoc, Nesibe Peker, Arzu Akcay, Devrim Gozuacik

**Affiliations:** ^1^ Koç University Research Center for Translational Medicine (KUTTAM), Istanbul, Turkey; ^2^ Yeni Yüzyıl University, School of Medicine, Private Gaziosmanpaşa Hospital, Department of Pathology, Istanbul, Turkey; ^3^ Koç University School of Medicine, Istanbul, Turkey; ^4^ Sabancı University Nanotechnology Research and Application Center (SUNUM), Istanbul, Turkey

**Keywords:** autophagy, dormancy, recurrence, relapse, cancer, metastasis

## Abstract

Metastasis and relapse account for the great majority of cancer-related deaths. Most metastatic lesions are micro metastases that have the capacity to remain in a non-dividing state called “dormancy” for months or even years. Commonly used anticancer drugs generally target actively dividing cancer cells. Therefore, cancer cells that remain in a dormant state evade conventional therapies and contribute to cancer recurrence. Cellular and molecular mechanisms of cancer dormancy are not fully understood. Recent studies indicate that a major cellular stress response mechanism, autophagy, plays an important role in the adaptation, survival and reactivation of dormant cells. In this review article, we will summarize accumulating knowledge about cellular and molecular mechanisms of cancer dormancy, and discuss the role and importance of autophagy in this context.

## Introduction

Cancer is the cause of death for millions of people every year, hence it’s one of the most devastating disease. Detection and diagnosis at early stages of cancer remarkably improve the chance of cure. However, the incidence of cancer continues to rise due various factors, including tobacco use, air pollution, obesity, increased life expectancy and cancer-causing infections. First approach in the treatment of cancer is usually surgical resection of the primary tumor, often followed by chemotherapy and/or radiotherapy. Besides, recent advances in targeted therapies and immunotherapies help to reduce the tumor burden. Thanks to high resolution diagnostic tools, advances in tumor ablation techniques, drug combinations, and targeted therapeutics, 5-year survival rates are improved for some cancer types, yet overall cancer survival rates for patients suffering from advanced disease are still low. A major reason for such discrepancy is the spread of cancer cells to organs other than the primary site and formation of the metastatic lesions. In other words, metastasis is among the leading causes of cancer-related deaths.

Metastasis of cancer to distant organs requires a sequential and complex chain of events. Cancer cells need to undergo several mutations and adaptations in order to gain motility and invasiveness, intravasate (migration into vessels), survive in the blood circulation and the lymphatics, extravasate, nestle and grow at secondary sites. Metastasis and survival of cancer cells at secondary sites are also affected by “the soil” in which tumor cells are seeded, namely the tumor microenvironment or stroma (1).

Mutations promoting epithelial-to-mesenchymal transition (EMT) greatly contribute to metastasis of cancer cells. Cells of normal tissues are tightly regulated by cell-to-cell and cell-to-matrix interactions. During cancerous transformation, epithelial cells may acquire mesenchymal cell-like properties, including loss of critical epithelial markers (e.g., E-cadherin, α-catenin), and expression of mesenchymal markers (e.g., N-cadherin and vimentin) (2). A transcriptional program orchestrates this transformation (e.g., ZEB1/2, Snail *etc*.) (3–5). Remodeling of epithelial junctions and cytoskeleton promotes motility and invasiveness of cancer cells (6). Cancer cells that are now motile and invasive, penetrate through the tissue extracellular matrix (ECM) and spread to lymph nodes and secondary sites through blood and lymph vessels. Seeding to metastatic sites and metastatic growth require reversal of this process, namely mesenchymal-to-epithelial transition (MET).

Advances in the last decade showed that in many tumor types, a small population of progenitor cancer cells, namely cancer stem cells (CSC), are responsible for the evolution and progression of the disease and metastasis (7, 8). Cancer cells and CSC might spread from primary tumors at various stages of tumor progression. These disseminated cells or clusters of cells (disseminated tumor cells, DTC) continue their evolution in their new tumor niches and they generally acquire genetic and epigenetic signatures that are different from the tumor of origin (9–13). Although aggressive proliferation of DTC might result in overt metastasis, latency periods lasting for months or even years were observed. During the latency period that spans the time between tumor formation and recurrence (also known as relapse), some cancer cells stay in a “dormant” state, a state of balanced proliferation or no proliferation at all (14, 15). At least some of these dormant cells have capacity to reactivate and form new metastatic lesions. Recurrent tumors were associated with drug resistance and aggressive behavior. So, most patients with recurrent disease show a very poor prognosis (16–18). For this reason, as an important mechanism contributing to tumor recurrence, cancer dormancy became a focus of attention in recent years.

**Figure 1:**
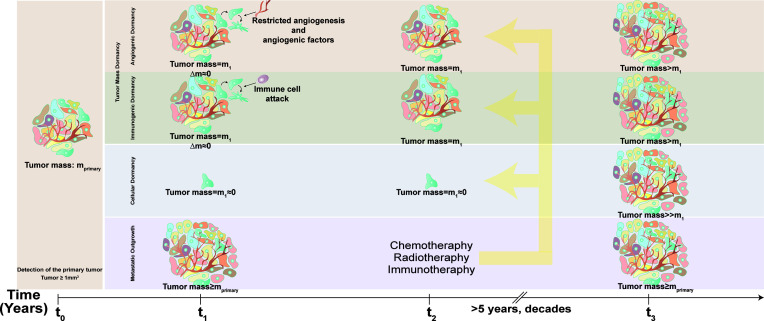
Time-dependent progression of metastasis and dormancy.** Conventional diagnostic tumor scans are able to detect tumors bigger than 1 mm^3^ (tumor mass = m_primary_). After diagnosis with cancer (time = t_0_), patient may undergo chemotherapy, radiotherapy or adjuvant therapy, yet dormant cells escape and become resistant to these treatments (time = t_2_), and awaken after years or even decades (time = t_3_). In tumor dormancy, tumor mass (m_1_) stagnates due to limited neovascularization and constant immune cell attack that balance tumor cell demise and proliferation. After the latency period, dormant tumor cells awaken and lead to tumor outgrowth (tumor mass>m_1_). In cellular dormancy, cancer cells hibernate as single cells or small clusters (tumor mass = m_1_≈0) and lead to massive tumor growth (tumor mass≥m_primary_) following exit from dormancy.

There are two major mechanisms of cancer dormancy, namely, tumor mass dormancy and tumor cell dormancy (or cellular dormancy) (**Figure 1**). In tumor mass dormancy, proliferation of tumor cells counterbalanced by cellular demise and the tumor mass is preserved to a certain extent. A reason for limited tumor growth is hypoxia and inefficient nutrient supply due vascularization defects (angiogenic dormancy). Trimming of tumor cells by the cells of the immune system is another mechanism limiting tumor growth and expansion (immunological dormancy). On the other hand, cellular dormancy involves transition to a quiescent, cell cycle-arrest state, while cells retain the capacity to perpetuate neoplastic behavior when reactivated. In this review, we will mainly focus on the role of autophagy in cellular dormancy.

## Autophagy and Cancer Dormancy

### Mechanisms of Mammalian Autophagy

Autophagy activation was reported as a novel characteristic of dormant cells in different tumor types (19). Three major types of autophagy were described: Macroautophagy, chaperone-mediated autophagy (CMA) and microautophagy. Although a clear connection between cancer and CMA was established (20), according to our knowledge, so far no study directly connecting CMA to cancer dormancy was published. Similarly, microautophagy was not studied in this context either. On the other hand, the number of studies implicating a role for macroautophagy in cancer dormancy continues to increase. Macroautophagy (autophagy herein) is an evolutionarily conserved catabolic process and an important stress response in all eukaryotic cells. Activation of autophagy leads to the clearance of various cellular components, including damaged organelles (e.g., mitochondria) as well as unfolded proteins and abnormal protein aggregates. As such, autophagy helps cells to combat stress, thereby contributes to survival. Mechanisms orchestrating autophagy activation, autophagic vesicle (autophagosome) formation and autophagic degradation were studied in detail.

**Figure 2:**
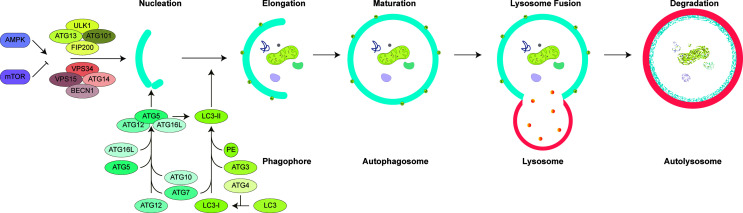
General mechanism of mammalian autophagy.** Autophagy is tightly controlled by the activity of AMPK and mTOR. Under nutrient deprivation, AMPK activates autophagy, yet mTOR inhibition is relieved. Subsequent activation of ULK1 and BECN1 complexes promotes formation of phagophore. ATG5-12-16L complex and ATG8 family protein LC3 are required for elongation and closure of phagophore. Fully mature double-layered autophagosome containing cargo molecules fuses with late endosomes and lysosomes. Autophagosomes and their cargo are degraded through lysosomal enzymes and recycled into cytosol for reuse.

Autophagic machinery primarily relies on the activity of ATG (autophagy-related) proteins (**Figure 2**). Following exposure to stress, activation of a core pathway involving ATG proteins leads to formation of double-membrane structures (phagophores) around target molecules and organelles. Phagophores eventually elongate and seal, forming closed vesicular structures called autophagosomes or autophagic vesicles. Autophagosomes fuse with late endosomes or lysosomes, to form autolysosomes. Lytic enzymes in the lumen of autolysosomes are responsible for the degradation of cargos carried by autophagosomes.

Autophagic activity is tightly controlled by protein complexes containing the mTOR kinase: mTORC1 and mTORC2 (21). These protein complexes are highly responsive to cellular cues, such as nutrient and growth factor availability, and in the active state, they work to inhibit autophagy (22). PKB/AKT pathway provides input from growth-related signals in order to regulate the mTOR complexes and autophagy. AMPK pathway, an energy sensor of the cell that monitors AMP/ATP ratios, comes into play when energy levels are low (23–25). While the mTORC1 has been documented to regulate autophagy directly, mTORC2 complex provides regulatory and feedback signals from insulin receptor phosphoinositide 3-kinase signaling (26–28). Under nutrient-rich conditions, mTORC1 keeps ULK1 and ATG13 in an inactive state. Nutrient deprivation leads to dephosphorylation of mTORC1 sites on ULK1 and ATG13 (24). ULK1 then autophosphorylates and phosphorylates its partner proteins ATG13 and FIP200 (29, 30). By this way, ULK1 activation initiates a cascade of events that promotes autophagosome formation.

Phagophore nucleation results from phosphorylation of lipids by the VPS34 lipid kinase complex (the class III PI3K, PI3KC3), BECN1 (Beclin-1), AMBRA1 and ATG14 (31, 32). Phosphorylation of inositol lipids on cellular membranes, such as ER membranes, leads to accumulation of phosphatidylinositol 3-phosphates (PI3P) (33). PI3P formation at membrane sites called omegasomes (or cradles), through recruitment of proteins with PI3P-binding domains, such as WIPI1/2 proteins and DFCP1 (34–36).

Proteins from the ubiquitin-like ATG8 family control elongation of phagophores through the activity of two key protein complexes. ATG12-ATG5–ATG16L1 complex facilitates coupling of ATG8 proteins, including MAP1LC3 (LC3) and GABARAPs, to phosphatidylethanolamine (PE) molecules on elongating membranes (37). Lipidated ATG8 proteins on autophagic membranes allow growth and closure of phagophore membranes (38, 39). Autophagy can be selective or non-selective. In the latter case, autophagy receptors, such as SQSTM1/p62, bridge between ATG8 proteins and ubiquitylated targets and direct them to autophagosomes. Hence, assessment of lipidation of ATG8s, especially LC3 lipidation, is a widely accepted as a powerful approach for monitoring autophagic activity (40, 41).

Mature autophagosomes are then transported along microtubules toward late endosomes and lysosomes. SNARE proteins facilitate fusion of autophagosomal outer membrane with endosomal or lysosomal membranes (42). Lysosomal acidic hydrolases degrade autophagic cargos into their building blocks (e.g. proteins into amino acids), which in turn are recycled to cytosol and used in the synthesis of new cellular components. As such, autophagy functions as a cellular degradation and recycling mechanism that allows cells to survive under stressful conditions.

Dysregulation of autophagy pathway is associated with various diseases, including cancer (43–45). In fact, autophagy plays an important yet context-dependent role at various stages of cancer progression and metastasis (45, 46). In early stages of cancer, control of ROS accumulation, prevention of DNA damage and genome instability require functional autophagic activity, eliminating damaged mitochondria and misfolded/aggregated proteins (47). Conversely, in established tumors and especially those tumors that grow relatively faster (e.g., tumors with K-RAS activation), tumor supporting role of autophagy is prominent. In this context, autophagy compensates for increased metabolic demands, originating from nutrient and energy deficiency, hypoxia and acidosis (48). Tumor stage-dependent dual role of autophagy might be explained in some models by hypoxia-triggered switches involving proteins, such as RAC3 (49). Moreover, autophagy was involved in various tumor progression- and metastasis-associated phenomena, including cell cycle regulation, stem cell behavior, extracellular matrix interactions, EMT, anoikis, tumor cell-stroma interactions, angiogenesis, immune responses and treatment resistance (50–54). In line with these observations, a number of autophagy genes and proteins show tumor suppressor or oncogenic activities (45, 55, 56).

In spite of the importance of autophagy in cancer formation and progression, contribution and molecular mechanisms of autophagy to cancer dormancy was not explored in detail in different cancer types and models. As summarized below, an increasing number of recent studies begins to provide evidence about a direct involvement of autophagy in cancer dormancy.

### Mechanisms of Cellular Dormancy

Cellular dormancy is characterized by a halt in cancer cell proliferation and entrance to a quiescence-like state. This non-proliferative state of existence may last for months or years. Moreover, no matter how long the dormant state is, some cells retain the capacity to reactivate and re-enter to a proliferative state (57–59). So, cellular dormancy is defined as a reversible mechanism.

Dormant cells usually reside and survive in the G0-G1 phase of the cell cycle. Hence, they lack proliferation markers (e.g., Ki67) as well as markers of apoptosis (e.g., active-caspases) and senescence (e.g., beta-galactosidase) (60–62). Not surprisingly, several changes in cell cycle regulatory molecules were observed. For instance, cyclin-dependent kinase (CDK) inhibitors p27^Kip1^ and p21^Cip1/WAF1^ controlled the non-proliferative state during hematopoietic stem cell dormancy (63). In another example, adhesion of lymphoma cells to bone marrow stromal cells resulted in cell cycle arrest, involving post-transcriptional regulation of Skp2, a component of p27^Kip1^ and p21^Cip1/WAF1^ containing SCF complex (64).

Another regulator of dormancy-associated cell cycle arrest was identified as the DREAM complex. The complex consists of p130 or p107 (Retinoblastoma-like pocket proteins), MuvB and E2F protein. MuvB was defined as a core component in the transcriptional regulation of cell cycle genes by the DREAM complex (65). In dormant cells, elevated p130 levels were shown to facilitate DREAM complex formation and regulate its transcriptional effects (65, 66). On the other hand, high levels of p107 were detected only in proliferating cells. Regulatory kinases DYRK1A and DYRK1B phosphorylated a subunit of MuvB, namely LIN52, and activated DREAM complex assembly during entry to the non-proliferative state (67). Additionally, these kinases stabilized p27^Kip1^ and induced cyclin D turnover, further contributing to the non-proliferative state (68, 69).

Mitogen-activated kinase (MAPK) pathway plays a central role in the regulation of dormancy. A number of dormancy-related factors and their cognate receptors were associated with a shift in the balance between proliferative ERK1/2 versus non-proliferative p38 MAPKs. Independent studies conducted in different cancer cell types, including breast, prostate, melanoma cells, supported the involvement of p38 pathway in cancer dormancy (70), and activation of this pathway contributed to the proliferation arrest in this context (71, 72). For instance, p38 kinases were stimulated by the activity of TGF-β2/TGFβRIII, which in turn supported dormancy of head and neck squamous cell carcinoma cells in the bone marrow (73). In addition, as a paracrine factor, secreted TGF-β2 from osteoblasts in the bone microenvironment contributed to prostate cancer dormancy through activation of p38 (74). Dormant cells secreted high levels of TGF-β2, creating an autocrine loop in the regulation of dormancy (75). In line with this, results revealed that proliferating cells have low TGF-β2 levels (75).

Urokinase plasminogen activator receptor (uPAR) pathway was described as another dormancy-associated pathway. In HNSCC, status of the uPAR was directly related with the dormancy capacity of cells *in vivo*. In this tumor type, interaction of uPAR with α5β1 integrin dictated formation of insoluble fibronectin fibrils and blocked the activation of p38 (76). Conversely, decreased uPAR levels were detected correlated with ERK1/2 pathway attenuation (76). Moreover, downregulation of uPAR inhibited focal adhesion kinase (FAK) phosphorylation and downstream Src activity, facilitating the dormant state *in vivo* (77, 78).

In addition to the HNSCC model, FAK and Src-related mechanisms were also studied in breast cancer dormancy. Activation of Src by CXCL2/CXCR4 signaling correlated with prolonged survival of DTC in the bone marrow niche *via* phosphoinositide 3-kinase (PI3K)/AKT pathway (79, 80). Interestingly, Src-assisted dormancy was secondary organ-dependent and its downregulation had no effect on lung metastasis of breast cancer cells (79, 80).

Bone morphogenetic proteins (BMP) were mainly involved in dormancy regulating tumor stroma interactions. Investigations on prostate cancer revealed that, BMP7 (bone morphogenetic protein 7) regulated dormancy of prostate cancer cells through affecting cancer stem cell population. This effect required BMPR2 (BMP receptor 2) expression and activation of dormancy-associated downstream signaling components, such as p38, p21 and the metastasis suppressor NDRG1 (81). In another study, bi-directional communication between tumor cells and stroma was revealed. Dormant prostate cancer cells, but not proliferative cells, secreted SPARC, a factor which stimulated BMP7 expression from bone marrow stromal cells, contributing to the maintenance of dormant phenotype (82). Another BMP protein, BMP4, was studied in the context of breast cancer. High levels of BMP4 expression correlated with entrance of cancer cells to a dormant state in the lung. In this organ, dormancy activation was associated with ALK2/3 and SMAD1/5 signaling (83). In this system, extracellular BMP antagonist DAND5 counteracted BMP4-assisted dormancy and promoted the proliferative state (84).

Dormancy-related signaling pathways lead to the activation of a specific gene regulatory program. For instance, BHLHE41 transcription factor was documented among the downstream effectors of p38-regulated dormancy in HNSCC (72). In another study, Kim et al. identified BHLHE41 and NR2F1 as key factors promoting ER positive breast cancer dormancy in an *in vivo* xenograft mice model (85). Importance of BHLHE41 to breast cancer dormancy was further confirmed in a 3D endosteal bone niche model containing bone marrow-derived cells and endothelial cells (86).

NR2F1 belongs to the NR2F family of cancer-related transcription factors (77). Dormant cancer cells were found to express high levels of NR2F1 in comparison to their proliferative counterparts (79, 87). Moreover, high NR2F1 and TGF-β2 expression were characterized as a dormancy signature in prostate cancer DTC (87). Moreover, transcription of another p38-regulated gene, SOX9, was directly controlled by NR2F1 binding elements in its promoter (87). NR2F1-SOX9 axis was also regulated by microenvironment-derived retinoic acid (RA) signaling and RARβ (87). In addition to other targets, NR2F1 promoted expression of the CXCL12 and its receptor CXCR4 and induced-cell cycle arrest in salivary adenoid cystic carcinoma cells (79).

Receptor tyrosine kinases, including TYRO3, AXL and MER, were critically involved in the dormancy phenotype of certain cancer types. For example, activation of AXL or TYRO3 receptor kinases by GAS6 secreted from osteoblast cells, contributed to the establishment of metastatic dormancy of prostate cancer cells in the bone marrow (75, 88, 89). In another example, dormant state was triggered in lymphoblastic leukemia cells by GAS6 to MER binding (90). On the other hand, AXL was found to be an important regulator of myeloid lineage-related gene expression and dormancy in myeloma cells (91).

Although generally considered as a pathway involved in cancer dissemination and metastasis (92), Wnt pathway was implicated in dormancy control in a context- and stimulus-dependent manner (93, 94). For instance, DKK1-dependent inhibition of Wnt3a signaling induced growth arrest and entry to dormancy (95). On the other hand, activation of Wnt5a pathway was responsible for the entrance of prostate cancer cells to a non-proliferative dormant state (94).

Overall, several cytokines, growth factors and signaling pathways involving kinases as well as transcription factors were identified as regulators of dormancy. Although we are far from having a complete picture, pathways regulating dormancy are being better defined. A summary of known proteins and pathways studied *in vitro* and *in vivo* were shown in **Tables 1** and **2** respectively, and the reader is referred to recent review articles about dormancy for further details (58, 191, 192).

**Table 1:** Summary of *in vitro* dormancy models and mechanisms.

	Proteins and Factors	Cell line	Tissue of Origin	Metastatic Target	Dormancy Tests	Effect on Dormancy	Dormancy Mechanism	Reference
**1**	Osteopontin	Nalm-6	ALL	Bone	Ki67 positivity, Fluorescent dye retaining (DiR), Drug resistance (Ara-C)	Induction	Tumor niche	(96)
**2**	Bcl-2	CD34-enriched primary AML	AML	N.D.	Drug resistance (Ara-C), Fluorescent dye retain (PKH26),	Induction	Tumor niche, apoptosis	(97)
**3**	FGF-2	T47D, MCF7	Breast	N.D.	Drug resistance (Taxotere)	Induction	Integrin and PI3K/Akt signaling	(98)
**4**	CK19	BT549, MDA-MB-231	Breast	N.D.	G0/G1 cell cycle arrest, Drug resistance (Cisplatin)	Induction	p38 signaling, ER stress	(99)
**5**	ΔNp63a	MCF7	Breast	N.D.	3D spheroid formation, G0/G1 cell cycle arrest, Drug resistance (Paclitaxel, Doxorubicin), Ki67and BrdU positivity	Induction	Wnt signaling	(100)
**6**	NR2F1-AS1	BT474	Breast	Lung	Ki67 positivity, Colony formation	Induction	Her2/neu and ER/PR hormone receptor signaling	(101)
**7**	p21	MCF10A	Breast	N.D.	BrdU positivity, G0/G1 cell cycle arrest	Induction	CDK2 signaling	(102)
**8**	miR-222/223	MDA-MB-231, T47D	Breast	Bone	G0/G1 cell cycle arrest, Drug resistance (Carboplatin), Stem-like phenotype (Oct4+)	Induction	CDKIs	(103)
**9**	IKKβ	MCF7	Breast	Bone, pelvic organs, lung	Colony formation, G0/G1 cell cycle arrest, Ki67 positivity	Induction	CDKIs, Stemness	(104)
**10**	BHLHE41, Wnt3, HBP1	MDA-MB-231	Breast	N.D.	3D spheroid formation	Induction	Tumor niche, p38/ERK signaling	(77)
**11**	IL1β	T47D, MCF7	Breast	N.D.	Drug resistance (Fulvestrant), colony formation, G0/G1 cell cycle arrest, p-p38/p-ERK1/2 ratio	Induction	Tumor niche, p38/ERK signaling, CDKIs	(105)
**12**	Fra-1	4TO7	Breast	N.D.	Drug resistance (Doxorubicin, Cyclophosphamide), G0/G1 cell cycle arrest, Stem-like phenotype (Sca-1+), Ki67 positivity	Induction	N.D.	(106)
**13**	LOXL2	MCF7	Breast	N.D.	Stemness (CD44 high/CD24low), 3D matrigel spheroid formation	Inhibition	EMT, Stemness	(107)
**14**	MLCK	D2.A1, D2OR, MCF7, MDA-MB-231	Breast	N.D.	3D spheroid formation	Inhibition	Integrin signaling, FAK, CDKIs	(108)
**15**	Src	D2.0R	Breast	Lung	Ki67 positivity, G0/G1 cell cycle arrest, 3D spheroid formation	Inhibition	ERK1/2 signaling, CDKIs	(109)
**16**	Profilin-1	MDA-MB-231	Breast	N.D.	3D matrigel spheroid formation	Inhibition	SMAD, FAK and ERK signaling	(110)
**17**	Parathyroid hormone-related protein (PTHrP)	MCF7	Breast	N.D.	RNA-seq dormancy associated gene downregulation (e.g. SOCS3, AMOT)	Inhibition	Ca+2 signaling	(111)
**18**	CXCL5	PyMT	Breast	Bone	Ki67 positivity	Inhibition	CXCL5/CXCR2 signaling	(112)
**19**	RhoA/RhoC	MCF-7, MDA-MB-231	Breast	N.D.	Ki67 positivity, Colony formation	Inhibition	ECM, JNK/SAPK signaling	(113)
**20**	Zeb1	D2A1, 67NR, 168 FARN	Breast	Lung, Bone, Adrenal gland	Fluorescent dye retaining (CFSE)	Inhibition	Immunogenic response	(114)
**21**	ZEB2	SW480	Colorectal	N.D.	Fluorescent dye retaining (PKH26)	Induction	EMT, CDKIs, Stemness	(115)
**22**	SDF-1α	HT-29, SW480	Colorectal	N.D.	Drug resistance (5-fluorouracil, irinotecan, oxaliplatin)	Induction	Tumor niche	(116)
**23**	IL-23/IL-23R	TE-1, ECA 109	Esophagus	N.D.	G0/G1 cell cycle arrest, Stem-like phenotype (CD133+), p21 and p16 expression, Radioresistance	Induction	Stat3/Wnt/Notch signaling	(117)
**24**	CXCL12, CXCL16 and CX3CL1	LN229, T98G	Glioblastoma	N.D.	Drug resistance (Temozolomide), Ki67 positivity, Fluorescent dye retaining (DiO), p-p38/p-ERK1/2 ratio	Induction	N.D.	(118)
**25**	PP2A	Primary Tumor stem-like cells (TSCs)	Glioblastoma	N.D.	G0/G1 cell cycle arrest, BrdU positivity	Inhibition	AKT and p53 signaling	(119)
**26**	Aurora kinase A (AURKA)	Hep2	Larynx	N.D.	Drug resistance (5-FU), G0/G1 cell cycle arrest,	Induction	CDKIs	(120)
**27**	miR-122	HCC-BCLC9 stem-like cell	Liver	N.D.	G0/G1 cell cycle arrest, p-p38/p-pERK1/2 ratio	Induction	Smad-independent TGF-β signaling, FOXO1, FOXO3A, MYC and AKT signaling	(121)
**28**	p53	A549, H460	Lung	N.D.	3D matrigel spheroid formation, Drug resistance (5-FU), G0/G1 cell cycle arrest, EdU positivity	Induction	TGF-β/smad-slug signaling, CDKIs, stemness	(122)
**29**	3D fibrin matrix stiffness	B16F10	Melanoma	N.D.	Ki67 positivity, PCNA positivity, G0/G1 cell cycle arrest	Induction	Integrin signaling, FAK, CDKIs	(123)
**30**	(TRP)-2	Prominin-1 (CD133)+ RET transgenic primary mouse melanoma cells	Melanoma	Bone	Ki67 and PCNA positivity	Induction	Immunogenic response	(124)
**31**	IGF2	AXT	Osteosarcoma	N.D.	G0/G1 cell cycle arrest, Ki67 positivity, Drug resistance (Adriamycin)	Induction	PI3K/Akt pathway, Autophagy	(125)
**32**	Dyrk1A	iOvCa147E2 and HEY	Ovary	N.D.	Cell cycle analysis by FACS, Drug resistance (Carboplatin)	Induction	CDKIs	(126)
**33**	AKT	OVCAR3, primary Epithelial ovarian cancer (EOC)	Ovary	N.D.	Ki67 and BrdU positivity, G0/G1 cell cycle arrest, 3D sphere formation	Inhibition	AKT signaling, CDKIs	(127)
**34**	NUP62	TOV112D	Ovary	N.D.	G0/G1 cell cycle arrest, Drug resistance (Cisplatin)	Inhibition	Nuclear pore architecture	(128)
**35**	TBK1	FGβ3	Pancreas	N.D.	Drug resistance (Erlotinib), 3D spheroid formation	Induction	Integrin signaling, αvβ3-KRAS-NF-κB axis	(129)
**36**	AKT	AsPC-1	Pancreas	N.D.	BrdU positivity, Drug resistance (5FU, SN38), G0/G1 cell cycle arrest, 3D matrigel spheroid formation	Inhibition	Tumor metabolism	(130)
**37**	Axl	PC3, DU145	Prostate	Bone	BrdU and Ki67 positivity, Fluorescent dye retaining (DiD)	Induction	TGFβ signaling	(75)
**38**	IRF7	RM1	Prostate	Bone, Lung	Fluorescent dye retaining (PKH26)	Induction	Immunogenic response, Type I IFN pathway (IFNAR)	(131)
**39**	MLCK	LuCaP 86.2, 92, and 93	Prostate	N.D.	Ki67 positivity	Inhibition	ECM, TGFβ signaling	(132)

**Table 2:** Summary of *in vivo* dormancy models and mechanisms.

	Proteins and Factors	Cell line	Tissue of origin	Metastatic target	Dormancy tests	Effect on dormancy	Dormancy mechanism	Reference
**1**	Notch3 ICD	MOLT-3, MICOL-14	ALL, Colorectal	N.D.	Xenograft tumor formation	Inhibition	Tumor niche	(133)
**2**	CXCL10	DA1-3b cell line	AML	Bone, spleen and liver	Allograft tumor formation ratio	Induction	Immunogenic response	(134)
**3**	ILK	J82, JB-V	Blader	N.D.	Xenograft tumor formation, Ki67 positivity	Inhibition	ECM	(135)
**4**	MMP2	Dunn, LM8	Bone	Liver, kidney, lung	Allograft tumor formation, 3D matrigel spheroid formation	Inhibition	Integrin signaling	(136)
**5**	miR-200b/200a/429 cluster	RJ345	Breast	Lung	3D matrigel spheroid formation, Xenograft tumor formation	Induction	EMT	(137)
**6**	POSTN	MDA-MB-231, T4-2	Breast	Bone, Brain, Lung	Kİ67 positivity, Allograft tumor formation	Induction	Angiogenesis, Tumor niche	(138)
**7**	MSK1	T47D	Breast	Bone	Ki67 and BrdU positivity, Xenograft tumor formation	Induction	p38 signaling, GATA3/FOXA1 axis	(139)
**8**	LPA1	4T1, MDA-MB-231T	Breast	Liver, Lung	Xenografted tumor formation, Ki67 positivity	Induction	p38, FAK, PLCβ signaling	(140)
**9**	Int2/Fgf3	MMTV(LA)-Induced Mammary Tumor	Breast	N.D.	Xenograft tumor fomation, BrdU positivity	Induction	Wnt signaling	(141)
**10**	Notch-2	MDA-MB-231	Breast	Liver	Ki67 and phospho-Histone H3b positivity, Drug resistance (Doxorubicin), Xenograft tumor formation, Stem-like phenotype (Sca1, CD34+)	Induction	Tumor niche, Notch signaling	(142)
**11**	TIE2	MCF7, 4T1	Breast	Bone	G0/G1 cell cycle arrest, Xenograft tumor formation, Drug resistance (5-FU)	Induction	Tumor niche, CDKIs	(143)
**12**	CXCR4	MDA-MB-231	Breast	Lung	G0/G1 cell cycle arrest, Ki67 positivity, Xenograft tumor formation	Induction	Tumor niche	(144)
**13**	Ron	PyMT-MSP	Breast	Lung	Allograft tumor formation	Induction	Immunogenic response	(145)
**14**	Fbxw7	E0771 and MDA-MB-231	Breast	Bone	Xenograft tumor formation, Allograft tumor formation, Drug resistance (Paclitaxel), Fluorescent dye retaining (PKH26), G0/G1 cell cycle arrest, 3D spheroid formation, Ki67 positivity	Induction	N.D.	(146)
**15**	BHLHE41, NR2F1	MCF7	Breast	N.D.	Xenograft tumor formation	Induction	N.D.	(85)
**16**	HSP27	MDA-MB-436	Breast	N.D.	Xenograft tumor formation, Ki67 positivity	Inhibition	Angiogenesis	(147)
**17**	Pfkfb3	D2.OR, D2.A1 cells	Breast	Lung	Xenograft tumor formation, 3D matrigel spheroid formation	Inhibition	Autophagy	(148)
**18**	FGFR1	Wnt1/ iFGFR1-driven breast cancer cell	Breast	N.D.	Xenograft tumor formation, Ki67 positivity	Inhibition	EGFR signaling	(149)
**19**	HER2/neu	MMTV-rtTA;TetO-NICD1 cells	Breast	N.D.	Colony formation, Tumor formation	Inhibition	Notch signaling	(150)
**20**	miR-205, miR-31	MDA-MB-231	Breast	Bone, brain and lung	Xenograft tumor formation, 3D sphere formation	Inhibition	Tumor niche, UBE2N/Ubc13 signaling	(151)
**21**	ROCK1	MDA-MB-231	Breast	N.D.	Xenograft tumor formation	Inhibition	Tumor niche	(152)
**22**	VCAM1	SCP6, TM40D, MCF7, CN34, MDA-MB-435	Breast	Bone	Xenograft tumor formation	Inhibition	Tumor niche, Integrin and NFκB signaling	(153)
**23**	Coco	4TO7, 4T1	Breast	Bone, Brain, Lung	Ki67 and EdU positivity, Allograft tumor formation, Fluorescent dye retaining (PKH26), 3D spheroid formation	Inhibition	Tumor niche, SMAD pathway, Stemness	(84)
**24**	Angiopoietin-2	MCF7	Breast	N.D.	3D matrigel spheroid formation, Xenograft tumor formation, p-p38/p-ERK 1/2 ratio,	Inhibition	Tumor niche	(154)
**25**	IRF7	4T1	Breast	Lung	Xenograft tumor fromation, Drug resistance (Methotrexate, Doxorubicin),	Induction	Immunogenic response, Type I IFN pathway (IFNAR)	(155)
**26**	ERK/p38	MDA-MB-231, MCF7, Hep3, M24met	Breast, Head and neck, Melanoma	N.D.	Xenograft tumor formation on CAM (Chorioallantoic membrane)	Induction	p38/ERK and Integrin signaling	(70)
**27**	H2BK, Eph receptor A5 (EphA5), Angiomotin	MDA-MB-436, KHOS-24OS, T98G, SW872	Breast, osteosarcoma, glioblastoma, liposarcoma	N.D.	Xenograft tumor formation	Induction	Angiogenesis	(156)
**28**	CUL4B	Patient-derived tumor organoid (PDOs) cell, HT29 and HCT116	Colorectal	Liver, Lung	3D matrigel spheroid formation, Xenograft tumor formation	Induction	Epigenetic alteration, Stemness	(157)
**29**	TSP-1, EGFR	U87-MG, T98G	Glioblastoma	N.D.	Xenograft tumor formation, Drug resistance (Erlotinib, Cetuximab), 3D matrigel spheroid formation	Induction	Angiogenesis, EGFR signaling	(158)
**30**	Tissue factor (TF)	U373	Glioblastoma	N.D.	Xenograft tumor formation, Ki67 positivity	Inhibition	Immunogenic response	(159)
**31**	miR-190	T98G, KHOS-24OS	Glioblastoma, Osteosarcoma	N.D.	Ki67 positivity, Xenograft tumor formation	Induction	Immunogenic response, antigen presenting	(160)
**32**	PRRX1	Cal-27, SCC-9	Head and neck	N.D.	Xenograft tumor formation	Induction	EMT, TGF-β and p38 signaling	(161)
**33**	α5β1 Integrin	HEp3	Head and neck	N.D.	Xenograft tumor formation on CAM (Chorioallantoic membrane), G0/G1 cell cycle arrest	Induction	p38 and Integrin signaling	(162)
**34**	ATF6a	D- and T-variant of HEp3	Head and neck	N.D.	Xenograft tumor formation on CAM (Chorioallantoic membrane)	Induction	UPR, Rheb-mTOR and MKK6/p-38 axis	(163)
**35**	BHLHE41	Hep3	Head and neck	N.D.	Xenograft tumor formation	Induction	p53, c-Jun signaling	(72)
**36**	Aurora kinase A (AURKA)	Hep2	Head and neck	Lung	Xenograft tumor formation, G0/G1 cell cycle arrest, Ki67 positivity	Inhibition	FAK/PI3K/Akt signaling	(164)
**37**	TGFβ2	Hep3	Head and neck	Lung, Bone	p-p38/p-ERK ratio, Xenograft tumor formation	Induction	SMAD pathway, CDKIs	(73)
**38**	NR2F1	Hep3	Head and neck	Spleen, Lung	Ki67 positivity, Xenograft tumor formation	Induction	Epigenetic alteration, Retinoic acid pathway, Stemness	(87)
**39**	Fibrinogen fibrils	HEp3	Head and neck	N.D.	Xenograft tumor formation on CAM (Chorioallantoic membrane)	Induction	p38/ERK and Integrin signaling	(76)
**40**	MYC	LAP-tTA Tet-o-MYC cells	Liver	N.D.	Ki67 positivity, Xenograft tumor formation	Inhibition	N.D.	(165)
**41**	YAP/TEAD	PC-9	Lung	N.D.	Xenograft tumor formation, Drug resistance (Osimertinib+ Trametinib)	Induction	EMT, Evasion of apoptosis	(166)
**42**	PAX5	Raji	Lymphoblastoid	N.D.	EdU, Fluorescent dye retaining (CFSE), G0/G1 cell cycle arrest, Xenograft tumor formation, Drug resistance (Etoposide, Daunorubicin)	Inhibition	N.D.	(167)
**43**	KISS1	C8161.9	Melanoma	Lung, Bone, Kidney, Eye	Xenograft tumor formation	Induction	Ca^+2^ and AKT signaling	(168)
**44**	Sox2	B16F1, A375	Melanoma	N.D.	3D fibrin spheroid formation, Ki67, COUP-TF1 and BrdU positivity, G0/G1 cell cycle arrest, Drug resistance (Tazarotene, ATRA, Temozolomide, Cisplatin), Stemness (CD133+) Xenograft tumor formation	Induction	Retinoic acid pathway, STAT3 and p53 signaling, CDKIs	(169)
**45**	IFN-γ	B16, A375	Melanoma	N.D.	3D spheroid formation, Xenograft tumor formation, G0/G1 cell cycle arrest, PCNA positivity, Drug resistance (Methotrexate, paclitaxol)	Induction	ECM, IDO1/AhR-dependent p27 pathway STAT1 pathway	(170)
**46**	Angiostatin	B16F10	Melanoma	Lung	Xenograft tumor formation	Induction	N.D.	(171)
**47**	VEGF	B16F10	Melanoma	N.D.	Xenograft tumor formation	Inhibition	Angiogenesis	(172)
**48**	GILZ	B16F1, B16F1-GM-CSF	Melanoma	Brain	Stemness (CD133, CD24 positivity), Xenograft tumor formation, G0/G1 cell cycle arrest	Inhibition	FOXO3A signaling, CDKIs	(173)
**49**	EET	LLC, B16F10,T241	Melanoma, Sarcoma	Lung, axillary lymph nodes, liver and kidney	Allograft tumor formation	Inhibition	Tumor niche, angiogenesis	(174)
**50**	TIMP-1 and TIMP-2	MLS 402-91 and primary human myxoid liposarcoma	Myxoid liposarcoma	N.D.	Xenograft tumor formation	Induction	Angiogenesis, lipogenesis	(175)
**51**	LTBP2	HONE1-2, NP460	Nasopharynx	N.D.	Colony formation, 3D matrigel spheroid formation, Xenograft tumor formation	Induction	N.D.	(176)
**52**	miR-34a, miR-93, and miR-200c	Saos-2, MG-63	Osteosarcoma	Lung	Ki67 positivity, Xenograft tumor formation	Induction	Angiogenesis, EMT	(177)
**53**	ARHI (DRAS3)	SKOv3, Hey	Ovary	N.D.	Xenograft tumor formation, Colony formation, PCNA positivity	Induction	Angiogenesis, Epigenetic alterations	(178)
**54**	VEGF, IL8 and IGF-1	SKOv3, OVCAR8	Ovary	N.D.	Xenograft tumor formation	Induction	Autophagy, ERK and AKT signaling	(179)
**55**	ARHI (DIRAS3)	SKOv3	Ovary	N.D.	Colony formation, Xenograft tumor formation	Induction	PI3K and TSC1/2 signaling, autophagy	(180)
**56**	MED12	HO8910 and SKOV3	Ovary	N.D.	Xenograft tumor formation, Colony formation, G0/G1 cell cycle arrest, Drug resistance (paclitaxel, gemcitabine, topotecan, and 5-FU)	Inhibition	EGFR signaling	(181)
**57**	CXCR4	A2780, SKOv-3	Ovary	N.D.	Drug resistance (cisplatin, doxorubicin, paclitaxel), Xenograft tumor formation, Colony formation	Inhibition	Tumor niche, EMT	(182)
**58**	IGF1	AsPC-1, MIA PaCa-2	Pancreas	N.D.	Allograft tumor formation, Xenograft tumor formation, G0/G1 cell cycle arrest, Ki67 positivity	Induction	IGF1/IGF-1R/AKT/XIAP signaling axis	(183)
**59**	IL8	R254, H6c7-kras, Panc1	Pancreas	N.D.	Ki67 positivity, p-P38/p-PERK1/2 ratio, Xenograft tumor formation	Induction	Tumor niche, Cytokine signaling	(184)
**60**	15-LOX-2	DU145, PC-3	Prostate	N.D.	Xenograft tumor formation, G0/G1 cell cycle arrest	Induction	Angiogenesis	(185)
**61**	MERTK	PC3, C4-2B	Prostate	Bone	Xenograft tumor formation, G0/G1 cell cycle arrest, Ki67 positivity	Induction	p38/ERK signaling, Stemness	(186)
**62**	BMP-7	PC3mm	Prostate	Bone	Xenograft tumor formation, Stemness (CD24low/CD44high/CD133high), Fluorescent dye retaining (DiD)	Induction	p38 signaling, SPARC/BMP7/BMPR2 axis, CDKIs	(82)
**63**	BMP7	PC3 mm, C4-2B	Prostate	Bone	3D sphere formation, Xenograft tumor formation, Stemness (CD24-/CD44+/CD133+)	Induction	BMPR2/NDRG1/P38 axis	(187)
**64**	GDF10, TGFβ2	C4-2B4	Prostate	Bone	Ki67 positivity, Xenograft tumor formation, p-p38/p-ERK1/2 ratio	Induction	Tumor niche, TGFβRIII-p38-pS249/T252 RB signaling axis	(74)
**65**	Axl	PC3, C42B	Prostate	Bone	Xenograft tumor formation	Induction	Tumor niche, platelet aggregation and activation	(188)
**66**	Axl, Tyro3	PC3, Du145	Prostate	Bone	Xenograft tumor formation	Induction	Tumor niche	(89)
**67**	Anxa2	PC3	Prostate	N.D.	Xenograft tumor formation, G0/G1 cell cycle arrest	Induction	Tumor niche	(90)
**68**	TBK1	PC3, C4-2B	Prostate	Bone	Xenograft tumor formation, Drug resistance (Taxotere)	Induction	Tumor niche, mTOR signaling	(189)
**69**	Wnt5a	PC3, C4-2B	Prostate	Bone	Fluorescent dye retaining (DiD), Xenograft tumor formation, Drug resistance (Docetaxel), G0/G1 cell cycle arrest, Ki67 positivity	Inhibition	Tumor niche, Wnt5a/ROR2/SIAH2 signaling axis, CDKIs	(94)
**70**	Angiostatin	PC-3, Colon A, MDA-MB-231; Lewis Lung, T241, M5076	Prostate, Colon, Breast, Lung, Fibrosarcoma, Sarcoma	N.D.	Xenograft tumor formation, Ki67 staining	Induction	Angiogenesis	(190)
**71**	NR2F1	SACC-83, SACC-LM	Salivary gland	Lung	G0/G1 cell cycle arrest, Xenograft tumor formation	Induction	CXCL12/CXCR4 signaling	(79)

### Role of Autophagy in the Context of Cancer Dormancy

In addition to recycling long-lived proteins, autophagy plays a key role in the management of energy crisis, control of reactive oxygen accumulation through destruction of damaged mitochondria, and in the elimination of unfolded and misfolded proteins. Studies in the last decade indicated that autophagy is involved in various stages of cancer formation and progression (193–197). As mentioned previously, autophagy plays a role in various events leading to tumor cell survival, resistance to treatment and metastasis. Hence, autophagy emerges as one of the critical determinants of the dormant state. In fact, several independent studies using cancer cells-derived from a wide variety cancer types, including breast, ovary, gastrointestinal tract, pancreas and bone cancers and their respective mice tumor or xenograft models showed that, autophagy is highly active in dormant cancer cells (125, 148, 180, 198–200). Some of these observations were even supported by the analysis of patient-derived tissue samples (201), yet molecular details of how and why autophagy contributes to the dormant phenotype are not well known. In this section, we will overview the current literature on autophagy-dormancy connection.

### Autophagy-Dormancy Connection: Experimental Evidence

Studies using different experimental set-ups, different cancer cell types and models revealed that, malignant cells entering a non-proliferative, dormancy-like but reversible cycle arrest state showed increased autophagic activity (**Table 3**) (148, 180, 199). In this context, dormant cancer cells were more sensitive to autophagy inhibition compared to their proliferating counterparts and inhibition of autophagy was lethal in most cases. Moreover, inhibition of autophagy in dormant cancer cells changed their metastatic behavior *in vivo* in mice (148, 199).

**Table 3:** Dormancy models with documented autophagic activity.

	Dormancy activating conditions	Cell line	Cancer type	Status of autophagy	References
**1**	Re-expression of DIRAS3/ARHI	SKOv3	Ovarian cancer	Activated	(180, 202, 203)
**2**	Akt1/2 inhibition	Ascites-derived primary human cancer cells	Ovarian cancer	Activated	(127, 204)
**3**	LKB1	Ascites-derived primary human cancer cells	Ovarian cancer	Activated	(205)
**4**	Farnesyltransferase inhibitors (FTIs)	MCF7	Breast cancer	Activated	(113)
**5**	Hypoxia/Re-oxygenation	MDA-MB-231	Breast cancer	Activated	(206)
**6**	Adriamycin-(ADR-) treatment	Neu-derived mammary cancer cells/mice model (FVBN202 mice)	Breast cancer	Activated	(207)
**7**	ECM	D2.A1, D2.0R	Breast cancer	Activated	(199)
**8**	Pfkfb3	D2.A1, D2.0R	Breast cancer	Activated	(148)
**9**	SYK inhibitor, R406	4T1	Breast cancer	Activated	(208)
**10**	SLC31A1 orT etrathiomolybdate (TM)	Panc‐1, MiaPaCa‐2	Pancreas cancer	Activated	(200)
**11**	IGF2 or Insulin	c-MYC in bone marrow stromal cells derived from Ink4a/Arf knockout mice cells (AXT)	Osteosarcoma	Activated	(125)
**12**	KIT/PDGFRA inhibitor, imatinib	GIST-T1	Gastrointestinal stromal tumor (GIST)	Activated	(198)

For instance, in gastrointestinal stromal tumor (GIST) cells, treatment with a KIT/PDGFRA inhibitor, imatinib, induced a dormancy-like quiescent state during which cells entered cell cycle arrest through accumulation of the cell cycle inhibitor p27 (198). Autophagy activation was observed under these conditions, and inhibition of autophagy using a genetic or chemical (chloroquine or quinacrine treatment) approach resulted in the loss of cell viability, and increased the anti-tumor efficacy of imatinib in *in vitro* and *in vivo* tests.

Contribution of autophagy to ovarian cancer dormancy was studied in detail. DIRAS3 (or ARHI) is a maternally imprinted tumor suppressor that is frequently downregulated in breast and ovarian cancers (209, 210). Re-expression of DIRAS3 in cancer cells robustly induced autophagy (180, 202, 211). Interestingly, although DIRAS3 expression resulted in the apoptotic death of cancer cells in culture (203), it promoted a dormancy-like state *in vivo* (180, 202). Re-expression of DIRAS3 in a Tet-inducible manner, stimulated autophagy in ovarian cancer xenografts, and led to a reversible inhibition of tumor growth and entry to a dormant state. Downregulation of the tumor suppressor was sufficient for the establishment of overt metastatic tumors. In this model, inhibition of DIRAS3-induced autophagy by chloroquine (a lysosomal autophagy inhibitor) reduced tumor growth, further underscoring the importance of autophagic activity to DIRAS3-related dormancy (202).

In other ovarian cancer studies, ascites-derived primary cancer cells from patients with high-grade serous ovarian cancer and ovarian cancer cell lines were used. Treatment of these cells with an allosteric AKT inhibitor Akti-1/2 induced a dormancy-like cytostatic response, and under these conditions, autophagic activity was significantly increased (204).

The role of autophagy in dormancy was studied in detail in breast cancer cell culture and animal models. Autophagy activation was observed during a dormancy-like arrest state of MCF7 breast cancer cells that were cultured with farnesyl transferase inhibitors (FTIs) (212). In MDA-MB-231 breast cancer cells, repetitive long-term hypoxia/reoxygenation cycles resulted in a low proliferation state and dormancy-like reversible cell cycle arrest (206). In another study, a dormancy-like state was induced by an adriamycin- (ADR-) treatment *in vitro* regimen using breast cancer cells from a Neu-driven cancer mice model (FVBN202 mice). Autophagy activation was also observed in this model of dormancy (207).

Other groups used two breast cancer cell lines derived from murine mammary hyperplastic alveolar nodules for modeling dormant versus proliferation states of this cancer type. D2.A1 cells were metastatic and D2.0R cells were dormancy-prone under certain growth conditions. In this system, autophagic activity of dormant D2.0R cells was found to be significantly higher than that of D2.A1 metastatic cells (148, 199). Both autophagosome and autolysosome numbers were increased, autophagy receptor (e.g. SQSTM1/p62) degradation was observed, indicating that autophagy in dormant cells was fully functional (148). In line with studies in other cancer types, dormant breast cancer cells were sensitive to autophagy inhibition whereas proliferative cells were resistant (199). Following tail vein injection to mice, most D2.0R cells stayed dormant in the lungs. Autophagy-related gene expression and autophagic activity in micrometastatic dormant lesions of D2.OR cells were observed higher as compared to the metastatic lesions of D2.A1 cells (148, 199).

Dormancy was also investigated in pancreatic duct adenocarcinoma (PDAC). In this cancer type, elevated levels of copper were associated with the degree of cancer progression. Interestingly, blockage of copper absorption by targeting the copper transporter 1 (SLC31A1) or usage of copper chelator tetrathiomolybdate (TM) inhibited proliferation of cancer cells and induced a dormancy-like arrest state (200). Under these conditions, autophagy was activated, and it was responsible for PDAC cell survival both *in vitro* and *in vivo* tests. Indeed, inhibition of autophagy caused an increase in *in vitro* cell death and decreased *in vivo* tumor burden. These results further provided evidence about the role of autophagy in the survival of dormant cells.

Dormancy in osteosarcoma following chemotherapy, has been associated with increased levels of IGF2 (125). Chronic exposure of osteosarcoma cells to IGF2 or insulin in combination with serum deprivation, successfully established an *in vitro* dormancy and drug-resistance model in osteosarcoma (125). Under these conditions, autophagy was activated.

Analysis of patient-derived samples provided further evidence about the importance of autophagy in cancer dormancy (201). In primary ovarian tumor tissue sections, LC3 localization in punctate structures was observed in only 21–23% of cases. In contrast, LC3 puncta, hence an upregulation of the autophagic activity was observed in more than 80% of tumor nodules found on the peritoneal surface of patients at second-look operations following primary chemotherapy. These results point out to a significant increase in autophagy in dormant ovarian cancer cells seeded in the peritoneum compared to primary tumor samples. These results underline the relevance and importance of experimental observations about dormancy-autophagy connection.

### Role of Autophagy in Dormancy Establishment, Dormant Cell Survival, and Reactivation

Autophagy controls dormant cell survival and behavior in many ways. In DIRAS3-induced ovarian cancer dormancy model, ARHI re-expression enabled SKOv3 ovarian cancer cells to remain dormant when they were grown in mice as xenografts (180). Reduction of ARHI levels in dormant cells caused xenografts to grow faster, and inhibition of ARHI-induced autophagy with chloroquine dramatically blocked regrowth of tumors.

In the D2.0R dormant and D2.A1 metastatic breast cancer cell models, autophagy was critical for the maintenance of the dormant phenotype in cancer cells and their survival. In 3D cultures, dormant D2.0R cells lost viability following treatment with autophagy inhibitors hydroxychloroquine, bafilomycin or 3-methyladenine, while non-dormant counterparts were not affected (199). Knockdown of autophagy genes Atg3, Atg7, p62 or FIP200, resulted in the outgrowth of dormant cells in 3D cell cultures. Moreover, Atg3-deficient D2.0R cells showed an increased capacity to create pulmonary tumors in mice (148). Similarly, in the ADR-induced dormancy model of Neu-driven breast cancer, mice that were *i.v.* injected with ADR-treated Atg5 knockdown cancer cells developed lung metastasis significantly sooner than those that were injected with wild-type dormant cells. As expected, a higher frequency of Ki67 positive, polyploid-like cells was observed in ADR-treated Atg5 knockdown mammary tumors (207). In line with these results, autophagy was downregulated in proliferating metastatic cells, but it was found to be necessary for a dormant-to-proliferative switch before the establishment of overt metastatic lesions (199). Consequently, treatment with autophagy inhibitors after the development of proliferative lesions (i.e. lesions that moved beyond the dormant-to-proliferative switch) had lesser impact on the metastatic burden (199).

These observations indicate that autophagy plays an active role in the initiation and maintenance of the dormant state, as well as during the switch from dormancy to a proliferative state.

### Role of Autophagy in the Clearance of Mitochondria and Regulation of Metabolism in Dormant Cancer Cells

Mitochondria are at the center of cellular energy metabolism control. A side product of oxidative phosphorylation is reactive oxygen species (ROS), and dysfunctional or damaged mitochondria are more prone to produce ROS. A life-threatening outcome of ROS accumulation at a cellular level is oxidation of building blocks such as proteins and lipids, as well as damage to DNA. A selective form of autophagy, mitophagy is a major mechanism that eliminates dysfunctional and damaged mitochondria and that ensures control of the mitochondrial mass in cells.

Cancer cells are able to stay in a dormant state for months or even years. Hence in dormant cells, in addition to elimination of unfolded/misfolded proteins and other building blocks, regulation of the mitochondrial mass and prevention of ROS accumulation should be of utmost importance for long-term survival and the preservation of reactivation capacity after transition to the proliferative state. Additionally, control of mitochondrial mass and function should be critical for metabolic reprogramming of dormant cells. Indeed, increased autophagic activity was associated with mitophagy in several models of cancer dormancy.

For instance, mitophagy was activated during DIRAS3-induced dormancy of ovarian cancer cells. Following DIRAS3 induction by the Tet-on system in ovarian cancer stable cell lines, TMRM uptake by mitochondria was decreased, indicating accumulation of depolarized mitochondria in these cells. Reduced TOM20 mitochondrial protein levels and mitochondrial mass as assessed through mitotracker staining were reported in dormant cells. As such, dormancy state was associated with a higher rate of mitochondrial depolarization, and mitophagy was increased as a mechanism to eliminate depolarized mitochondria and to limit ROS accumulation (213).

In the D2.0R breast cancer model of dormancy, autophagy protein LC3 was found to colocalize with mitochondria in cells growing in a matrix supporting dormancy. During mitophagy, PINK-assisted ubiquitylation of mitochondrial proteins by E3 ligases such as PARKIN prime mitochondria for mitophagic degradation. Indeed, accumulation of mitophagy-associated full length PINK and degradation of mitochondrial protein TOM20 was reported under these experimental conditions. Additionally, autophagy inhibition using HQ caused an accumulation of damaged mitochondria as well as ROS. Following suppression of the autophagic activity, dormant cells suffered from DNA damage, caspase-3 activation was prominent, and cells eventually died. Mitochondrial ROS scavengers prevented cell death, indicating that an important function of autophagy in dormant cells is the maintenance of healthy mitochondrial mass, hence limitation of ROS-induced damage (199). Similarly, in the TM-treated PDAC cell model of dormancy, inhibition of autophagy by CQ increased ROS accumulation and resulted in cell death, further showing that ROS limiting activity of autophagy is central to dormant cancer cell survival in different cancer models (200).

Autophagic and mitophagy activation during dormancy was associated with metabolic changes in cells. In the ovarian cancer dormancy model, induction of DIRAS3 resulted in a higher glycolytic rate and mitochondrial respiration rate was decreased (213). Indeed, ATP levels of were found to be attenuated in different models of dormancy (200, 205, 213). Moreover, in dormant cells, an increase in glucose and glutamate uptake was accompanied by extracellular lactate accumulation (213). Under these conditions, increased glucose uptake was coupled to an upregulation of glycolysis and glutaminolysis, and all these changes were autophagy dependent. In this context, blockage of these metabolic pathways resulted in decreased cell viability (213). In autophagic tumors *in vivo*, free valine, glycine, and alanine concentrations were increased at statistically significant levels, indicating that bulk degradation of proteins by autophagy was also accelerated and it further supported metabolic activities of dormant cancer cells (213).

### Molecular Mechanisms of Autophagy in Dormant Cells

To date, molecular mechanisms governing autophagy activation during dormancy and autophagy signaling pathways that are involved are largely unknown. Yet, studies on autophagy-dormancy connection provided hints about the involvement of certain autophagy-related proteins and pathways in the process.

Among the upstream signaling pathways regulating autophagy, inhibition of the PI3K/AKT pathway emerges as a common observation. In many reports, mTOR pathway that is downstream to AKT was shown to be inhibited in dormant cells. As mentioned above, mTOR is a central regulator of autophagy, and its inhibition correlates with autophagy activation in various systems (214–217).

In ovarian cancer dormancy model, DIRAS3 expression resulted in the inhibition of signaling through PI3K/AKT and Ras/MAP through enhancing internalization and degradation of the epidermal growth factor receptor. As a result, mTOR signaling downstream to the AKT pathway was also inhibited (180, 201). Indeed, downregulation the pathway by DIRAS3 resulted in a decrease in the activation of mTOR downstream pathway proteins, such as p70S6K and pS6, proteins that are involved in the regulation of cell size and protein synthesis (180). Autophagic activity was strongly stimulated under these conditions.

In line with the DIRAS3 model, ascites-derived ovarian cancer spheroids were in a dormant state that was associated with AKT downregulation and autophagy activation (127, 205). In fact, inhibition of the AKT pathway in ovarian cancer cells using specific inhibitors of the AKT kinase, namely Akti-1/2, was sufficient to direct the entry of cells to a dormant-like state (127). Downregulation of AKT and mTOR pathway was also observed in the osteosarcoma dormancy model (125) and breast cancer cells entering dormancy following exposure to long-term hypoxia/reoxygenation cycles (206). Similarly, mTOR and its downstream pathways were reported to be inhibited in the imatinib-induced GIST cell dormancy model (198).

Another key protein regulating autophagy activation is the energy sensor kinase AMPK. Increased AMP/ATP ratio correlates with problems in energy status of cells, and leads to the activation of AMPK (218). Hypoxia is another signal that can activate AMPK (219). Following activation, AMPK was shown to phosphorylate TSC2 and interfere with the activity of the GTPase RHEB, an activator of mTORC1 signaling; the net result is autophagy activation (220, 221). Another mechanism through which the kinase contributes to autophagy activation direct phosphorylation of the autophagy protein ULK1 (23, 24).

In dormant cells, intracellular ATP levels are decreased (74, 205, 213). Consequently, AMPK activation was reported in different experimental models of dormancy. For instance, in ovarian tumor cells, LKB1 and AMPK expression and activity were increased during spheroid formation and dormancy (205). The study showed that LKB1 (and possibly AMPK) was required for the survival of ovarian cancer cells in a dormant state. Moreover, AMPK activation in proliferating ovarian cancer cells caused them to enter cell cycle arrest (205). Similarly, an increase in AMPK activity was observed during DIRAS-3-induced dormancy of ovarian cancer cells (180) and chronic hypoxia-induced dormancy of breast cancer cells (206).

Transcriptional upregulation of autophagy-related genes was observed in dormancy models. Independent groups reported the upregulation of key autophagy genes, including LC3, ATG4, ATG5, ATG7 and BECN1, in dormant cells (148, 199). A mechanistic explanation on the transcriptional regulation of autophagy gene expression in the dormant state came from studies using ovarian cancer cells. During DIRAS3-induced dormancy, mTOR inhibition promoted translocation of transcription factors FOXO3a and TFEB to the nucleus (222). The end result was a FOXO3a-dependent upregulation of autophagy proteins ATG4 and LC3 and Rab7, a mediator of autophagosome-autolysosome fusion (222). Similar to FOXO3a, TFEB translocation to the nucleus activated transcription of various autophagy-related genes (223, 224).

Interestingly DIRAS3 itself was subject to transcriptional regulation downstream to the mTOR pathway (225). Under conditions of nutrient deprivation, mTOR inhibition resulted in the dissociation of E2F1 and E2F4 from the DIRAS3 promoter, leading to the proteasomal degradation of these transcription factors. Dissociation of E2F1 and E2F4 from DIRAS3 promoter allowed transcriptional upregulation of the gene and activated autophagy. On the other hand, another transcription factor, CEBPα, positively regulated DIRAS3 expression and autophagy. Hence, transcriptional loops involving DIRAS3 might contribute to further activate and sustain autophagy during nutrient deprivation and possibly during dormancy (225).

DIRAS3 was directly participating to autophagy regulating protein complexes. In fact, DIRAS3 was shown to stabilize the autophagy initiation complex consisting of VPS34 (PIK3C3), BECN1 and ATG14 (201). DIRAS3 binding to BECN1 destabilized BECN1-BCL2 inhibitory complexes, displaced BCL2 and allowed recruitment of BECN1 protein by autophagy-related VPS34 lipid kinase complex. Binding of DIRAS3 to BECN1 facilitated association of BECN1 with VPS34 and ATG14. DIRAS3 was also shown to directly bind VPS34. Altogether, DIRAS3 enhanced VPS34 lipid kinase activity that is required for autophagosome formation and autophagy activation (201). Moreover, DIRAS3-mediated stabilization of the initiation complex and subsequent autophagy activation was necessary for dormant cell survival after chemotherapy (201).

On the other hand in mice, knockdown of Atg7 but not Becn1 decreased numbers of tumors formed by dormancy-prone cells in a TGFβ-induced inflammatory background, indicating that requirement for Becn1 gene in dormancy-related autophagy and tumor cell survival might be tumor and cell type-dependent (199).

### Role of Autophagic Degradation in Dormancy

Data that was presented above show that autophagic activity is prominently higher in dormant cancer cells compared to their actively proliferating counterparts. Several studies provided clues about the nature of autophagy in this setting. In addition to an increase in autophagosome numbers, autolysosome formation and autolysosomal degradation was reported to be upregulated during dormancy. Autolysosomal degradation of the selective autophagy receptor p62/SQSTM1 as well as the LC3 protein itself was reported in many studies (148, 199). Inhibition of the autolysosomal activity by chemicals, such as chloroquine and its derivatives, changed the behavior of dormant cells, influenced cell survival, dormant cell reactivation and metastatic capacity. As explained above, mitochondria are among the targets of selective autophagic degradation during dormancy. Therefore, metabolic outcomes of autophagic activity in dormant cells might be attributed to mitophagy and non-selective autophagic degradation of cellular components, including long-lived proteins. So far, the role of selective autophagy in cancer dormancy is not well studied, and there are only a few reported examples.

A dormancy-related direct target of autophagy was identified using the D2.0R model of breast cancer dormancy. In this study, Pfkfb3 (6-phosphofructo-2-kinase/fructose 2,6-biphosphatase 3) was identified as a gene that was highly expressed in metastatic cells but downregulated in dormant cancer cells (148). Pfkfb3 is a key regulator of glycolysis rate in cells, and its expression was shown to promote metastatic tumor growth. An inverse correlation between dormancy-related autophagic activity and Pfkfb3 levels was observed. So, the role of autophagy in the degradation of Pfkfb3 protein was studied in dormant cancer cells. Indeed, Pfkfb3 protein accumulated when autophagic degradation was inhibited using CQ or autophagy gene knockdown. The protein was polyubiquitylated, and in this state, it directly interacted with the ubiquitin-associated domain (UBA) of the p62 protein. Strikingly, Pfkfb3 downregulation in metastatic D2.A1 breast cancer cells prevented their growth and delayed establishment of metastatic lesions. Conversely, autophagy inhibition and Pfkfb3 upregulation correlated with reactivation of dormant of D2.0R cells. The study showed that, although proteasomal degradation also contributes to the determination of protein’s half-life, selective degradation by autophagy is important in the control of Pfkfb3 protein levels in dormant cells. Hence, the study provides an example where tumor dormancy and recurrence rely on autophagic clearance of metabolic regulators (148).

Another autophagy target was identified among factors regulating EMT-MET (epithelial-mesenchymal and mesenchymal- epithelial transitions) during metastasis. SYK is a non-receptor tyrosine kinase mediating signaling events downstream to several transmembrane receptors, including the B-cell receptor (BCR). Decreased expression of SYK mRNA correlated with decreased survival in breast cancer patients (226). P-bodies are cytoplasmic foci containing mRNA, miRNA and mRNA-binding proteins, and they are involved in the regulation of mRNA half-life and translation control. During TGFβ-induced EMT, accumulation of P-bodies was observed. SYK concentrated in P-bodies, and SYK activity and autophagy was necessary for controlled clearance of P-bodies during MET and metastasis (208, 227). Hence in this system, SYK promoted removal of P-bodies through autophagy and supported activation dormant cancer cells, allowing initiation of cancer metastatic outgrowth (208).

There are possibly other direct or indirect targets of autophagy that are involved in dormancy maintenance and a dormant-to-proliferative switch. Further studies will reveal the identity of these key factors that are degraded by dormancy-associated autophagic activity.

### Treatment Responses, Dormancy, and Autophagy

Current cancer treatment approaches are usually unable to result in the total elimination of disseminated cancer cells and micrometastases. They even seem to create a selective pressure on cancer cells promoting their escape from cell death by entering to a dormant state. Since dormant cells are not actively proliferating, they are in general resistant to chemotherapy and radiotherapy approaches that mainly result in DNA damage and block cell division. A body of literature provide evidence about the role of autophagy in the treatment resistance of growing primary tumors and overt metastases (228–231). Since autophagic activity is increased in dormant cells, autophagy might be a contributing factor in the observed robustness of dormant cells when faced with anticancer insults.

A number of studies tested the contribution of autophagy to treatment resistance that is observed in dormant cells. First observation is some drugs that were utilized in order to create models of dormancy, they themselves induced autophagy upregulation in cells. For example, treatment of cancer cells with imatinib (198), farnesyltransferase inhibitors (212), AKT inhibitor (204), and adriamycin (207), resulted in the upregulation of autophagic activity. Usage of antimalarial lysomorphic inhibitors of autolysosomal activity, such as Chloroquine, Hydroxychloroquine or quinacrine, as a combination treatment along with chemotherapy agents blocked autophagy under these conditions and generally resulted in the death of dormant cells and even elimination of tumors (125, 180, 202, 207). Combination of chemotherapy with genetic approaches gave similar results as well (125, 202, 207). So, capacity to activate sustained autophagy in response to cancer therapies might be one of the critical factors favoring the selection of dormant cells. This “autophagy addiction” might be exploited for the elimination of disseminated dormant cells in patients. On the other hand, considering indications about the role of autophagy in the dormant-to-proliferative switch, inhibition of autophagy might promote reactivation of dormant cancer cells, leading them to reenter an active proliferative state that renders them again susceptible to antiproliferative cancer treatments. On the other hand, crizotinib, an ALK inhibitor was shown to further activate autophagy and trigger apoptosis of dormant ovarian cancer cells (232). Either way, all these studies underline the therapeutic potential of autophagy manipulation in the context of dormancy.

## Conclusion

Drug resistance and cancer dormancy are the two important causes of incurable metastatic disease that results in the loss of millions of lives from cancer-related deaths every year. Autophagy emerges as an integral part of the dormancy phenomenon. Autophagy activation was observed in dormant cells originating from different types of cancers, in cancer cellular models and animal models, as well as in patient-derived cells and tissues.

Autophagic activity was shown to confer survival advantage, treatment resistance and resilience to dormant cells. An important contribution of autophagy to dormant cell survival was related to the limitation of ROS accumulation. Autophagy is an important mechanism for the elimination of depolarized mitochondria, damaged peroxisomes and other organelles, as well as cytosolic long-lived proteins that are prone to aggregate and accumulate in the cytosol when exposed to excessive oxidative damage. Protection and preservation of the genetic material from ROS damage during long-lasting non-proliferative periods that may last for months or years, such as those observed during dormancy, is also an important challenge. For cells to preserve the reactivation capacity, dormant cells should be able to limit the number and extent of mutations they accumulate during periods of cell cycle arrest. Potency and efficacy of DNA repair pathways in dormant cells is not clear and further studies are required (233–235). At this point, studies in normal stem cell quiescence might give indications about the faith of DNA in cells that reside in long-term dormant periods. These studies indicated that in quiescent cells, DNA damage burden may even be higher in older cells than younger ones, and that repair process only begins following entry to cell cycle (236). Hence, these data underline the fact that, limitation of ROS accumulation by the autophagic activity contributes significantly to the survival of dormant cells.

Autophagy seems to play a critical role in the maintenance of dormant phenotype. Autophagy-deficient cells were not able to enter or stay in a dormant state compared to controls. Mechanisms through which autophagy controls the dormant phenotype are not clear. So far, only a few dormancy-related targets of autophagy were described. Pfkfb3 protein was identified as a target of selective autophagy in dormant cells. In fact, in addition to being a regulator of glycolysis, Pfkfb3 was shown to translocate to nuclei and its product fructose 2,6-biphosphate was shown to inhibit cell cycle inhibitor p27^Kip^ and activate cyclin D3, resulting in progression from the G1 phase to the S phase (237). Moreover, Pfkfb3 was also involved in the upregulation of CDK1 and Cdc25 expression promoting entry to mitosis (237). Therefore, selective targeting of key proteins involved in cell cycle by autophagy, such as Pfkfb3, may be an important function of autophagy in the entry to and maintenance of the dormant phenotype.

P-bodies were also reported as selective targets of autophagy in a dormancy context. Whether autophagic degradation control a general downregulation of P-bodies or whether it is selectively and deliberately targeting a cell cycle- and dormancy-relevant subset of mRNAs, miRNAs and/or proteins is not clear.

Characterization of a full list of selective autophagy targets during dormancy will allow a better understanding of the role and contribution of autophagic degradation to the dormant phenotype.

Mechanistic aspects of autophagy signaling during dormancy are being better understood. In fact, AKT and related growth factor pathways seem to emerge as an important regulators of autophagy activation in dormant cells. Downstream to AKT, mTOR pathway components are inhibited, resulting in the activation of autophagy proteins, including ULK1. A decline in ATP levels in dormant cells may activate the energy sensor systems LKB1 and AMPK and further inhibit the mTOR pathway through TSC2 phosphorylation and activate autophagy by phosphorylating ULK1. Deficiency of nutrients, such as amino acids, possibly contribute to inhibition of mTOR on the lysosomes (238). mTOR inhibition allows activation of factors FoxO3a and TFEB, that transcriptionally upregulate autophagy-related genes.

Although most components of the canonical autophagy pathways were reported to be involved in dormancy-related autophagy, some studies questioned the contribution of key proteins, such as BECN1. Others placed BECN1 containing complexes at the center of autophagy regulation in dormant cells. ATG5 and ATG7 were reported to be important as well. p62/SQSTM1 was degraded in several independent models of dormancy, indicating that it may be an important mediator of selective autophagy under these conditions. Whether other autophagy receptors contribute to the autophagy pathway in dormant cells need further investigation.

Inhibition of autophagy by drugs or genetic methods was reported to impede dormancy, affect cell survival, or lead cells to enter into a proliferative phase, in which cancer cells are more susceptible to be eliminated by common cancer therapeutic strategies. Hence, approaches involving promotion of dormancy or reactivation of dormant cells should both necessitate study and manipulation of autophagy. A detailed understanding of mechanisms, regulatory pathways and specific targets of autophagy in the context of dormancy will certainly contribute to a better management of metastatic and recurrent disease, and maybe allow one day total elimination of disseminated cells and micrometastases in all cancer patients.

## Author Contributions

DG designed the structure of the review and wrote and edited the review article. YA and NP wrote the review article and prepared the tables and figures. All authors contributed to the article and approved the submitted version.

## Funding

This work was supported by Scientific and Technological Research Council of Turkey (TUBITAK) grant number 216S489.

## Conflict of Interest

The authors declare that the research was conducted in the absence of any commercial or financial relationships that could be construed as a potential conflict of interest.
